# *N*,*N*′-Bis(3-chloro­phen­yl)succinamide

**DOI:** 10.1107/S1600536811010440

**Published:** 2011-03-26

**Authors:** B. S. Saraswathi, Sabine Foro, B. Thimme Gowda

**Affiliations:** a Department of Chemistry, Mangalore University, Mangalagangotri 574 199, Mangalore, India; b Institute of Materials Science, Darmstadt University of Technology, Petersenstrasse 23, D-64287 Darmstadt, Germany

## Abstract

The complete molecule of the title compound, C_16_H_14_Cl_2_N_2_O_2_, is generated by crystallographic inversion symmetry. The dihedral angle between the benzene ring and the NH—C(O)—C fragment is 32.8 (1)°. In the crystal, the molecules are linked by N—H⋯O hydrogen bonds into [100] chains.

## Related literature

For our study of the effect of substituents on the structures of *N*-(ar­yl)-amides, see: Gowda *et al.* (2000 ▶); Saraswathi *et al.* (2011 ▶), of *N*-(ar­yl)-methane­sulfonamides, see: Gowda *et al.* (2007 ▶) and of *N*-(substitutedphen­yl)-*p*-substituted-benzene­sulfonamides, see: Gowda *et al.* (2005 ▶). 
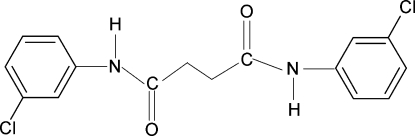


## Experimental

### 

#### Crystal data

C_16_H_14_Cl_2_N_2_O_2_
*M* *_r_* = 337.19
Monoclinic, 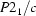

*a* = 8.3412 (8) Å
*b* = 9.6501 (9) Å
*c* = 9.5485 (9) Å
β = 91.319 (9)°
*V* = 768.39 (13) Å^3^
*Z* = 2
Mo *K*α radiation
μ = 0.43 mm^−1^
*T* = 293 K
0.40 × 0.20 × 0.20 mm

#### Data collection

Oxford Diffraction Xcalibur diffractometer with Sapphire CCD detector
Absorption correction: multi-scan (*CrysAlis RED*; Oxford Diffraction, 2009 ▶) *T*_min_ = 0.847, *T*_max_ = 0.919
2574 measured reflections
1535 independent reflections
1253 reflections with *I* > 2σ(*I*)
*R*_int_ = 0.009

#### Refinement

*R*[*F* ^2^ > 2σ(*F* ^2^)] = 0.037
*wR*(*F* ^2^) = 0.105
*S* = 1.07
1535 reflections
103 parameters
1 restraint
H atoms treated by a mixture of independent and constrained refinement
Δρ_max_ = 0.25 e Å^−3^
Δρ_min_ = −0.34 e Å^−3^

### 

Data collection: *CrysAlis CCD* (Oxford Diffraction, 2009 ▶); cell refinement: *CrysAlis RED* (Oxford Diffraction, 2009 ▶); data reduction: *CrysAlis RED*; program(s) used to solve structure: *SHELXS97* (Sheldrick, 2008 ▶); program(s) used to refine structure: *SHELXL97* (Sheldrick, 2008 ▶); molecular graphics: *PLATON* (Spek, 2009 ▶); software used to prepare material for publication: *SHELXL97*.

## Supplementary Material

Crystal structure: contains datablocks I, global. DOI: 10.1107/S1600536811010440/ds2100sup1.cif

Structure factors: contains datablocks I. DOI: 10.1107/S1600536811010440/ds2100Isup2.hkl

Additional supplementary materials:  crystallographic information; 3D view; checkCIF report

## Figures and Tables

**Table 1:** Hydrogen-bond geometry (Å, °)

*D*—H⋯*A*	*D*—H	H⋯*A*	*D*⋯*A*	*D*—H⋯*A*
N1—H1*N*⋯O1^i^	0.81 (2)	2.10 (2)	2.8946 (19)	166 (2)

Symmetry code: (i) 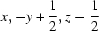.

**Fig. 1.**
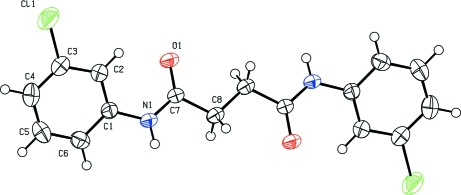
Molecular structure of (I), showing the atom labelling scheme and displacement ellipsoids are drawn at the 50% probability level.

**Fig. 2.**
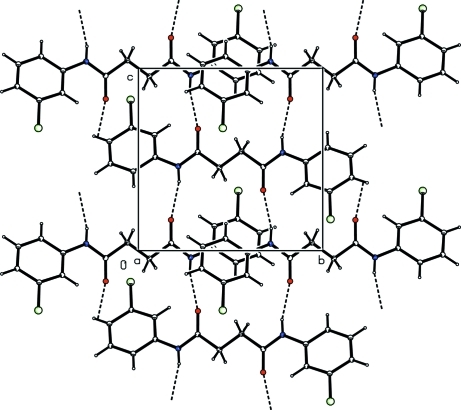
Molecular packing of (I) with hydrogen bonding shown as dashed lines.

## supplementary crystallographic information

### Comment

The amide and sulfonamide moieties are important constituents of many biologically significant compounds. As a part of studying the substituent effects on the structures of this class of compounds(Gowda *et al.*, 2000, 2005, 2007; Saraswathi *et al.*, 2011), in the present work, the structure of *N*,*N*-bis(3-chlorophenyl)-succinamide (I) has been determined (Fig.1). The conformations of N—H and C=O bonds in the C—NH—C(O)—C segments are *anti* to each other and the amide O atoms are *anti* to the H atoms attached to the adjacent C atoms. Further, conformations of the N—H bonds in the amide fragments are *anti* to the *meta*-chloro groups in the adjacent benzene rings, similar to the *anti* conformations observed with respect to the *ortho*-methyl groups in *N*,*N*-bis(2-methylphenyl)- succinamide (II) (Saraswathi *et al.*, 2011). The dihedral angle between the benzene ring and the NH—C(O)—CH_2_ segment in the two halves of the molecule is 32.8 (1)°, compared to the value of 62.1 (2)° in (II).

Further, C1—N1—C7—C8 and C1a—N1a—C7a—C8a segments in (I) are nearly linear and so also the C1—N1—C7—O1 and C1a—N1a—C7a—O1a segments, similar to those observed in (II). The torsion angles of C2—C1—N1—C7 and C6—C1—N1—C7 are -35.0 (3)° and 147.5 (2)°, in contrast to the values of -64.0 (4)° and 117.6 (3)° in (II).

The packing of molecules in the crystal linked by of N—H···O hydrogen bonds (Table 1) is shown in Fig. 2.

### Experimental

Succinic anhydride (0.01 mol) in toluene (25 ml) was treated drop wise with 3-chloroaniline (0.01 mol) also in toluene (20 ml) with constant stirring. The resulting mixture was stirred for one hour and set aside for an additional hour at room temperature for completion of the reaction. The mixture was then treated with dilute hydrochloric acid to remove unreacted 3-chloroaniline. The resultant solid *N*-(3-chlorophenyl)-succinamic acid was filtered under suction and washed thoroughly with water to remove the unreacted succinic anhydride and succinic acid. The compound was recrystallized to constant melting point from ethanol. The purity of the compound was checked by elemental analysis and characterized by its infrared and NMR spectra.

The *N*-(3-chlorophenyl)succinamic acid obtained was then treated with phosphorous oxychloride and excess of 3-chloroaniline at room temperature with constant stirring. The resultant mixture was stirred for 4 h, kept aside for additional 6 h for completion of the reaction and poured slowly into crushed ice with constant stirring. It was kept aside for a day. The resultant solid, *N*,*N*-bis(3-chlorophenyl)- succinamide was filtered under suction, washed thoroughly with water, dilute sodium hydroxide solution and finally with water. It was recrystallized to constant melting point from a mixture of acetone and chloroform. The purity of the compound was checked by elemental analysis, and characterized by its infrared and NMR spectra.

Rod like colorless single crystals used in the X-ray diffraction studies were were grown in a mixture of acetone and chloroform at room temperature.

### Refinement

The H atom of the NH group was located in a difference map and later restrained to the distance N—H = 0.86 (2) Å. The other H atoms were positioned with idealized geometry using a riding model with the aromatic C—H = 0.93Å and the methylene C—H = 0.97 Å. All H atoms were refined with isotropic displacement parameters (set to 1.2 times of the *U*_eq_ of the parent atom).

### Crystal data

**Table:**

C_16_H_14_Cl_2_N_2_O_2_	*F*(000) = 348
*M**_r_* = 337.19	*D*_x_ = 1.457 Mg m^−^^3^
Monoclinic, *P*2_1_/*c*	Mo *K*α radiation, λ = 0.71073 Å
Hall symbol: -P 2ybc	Cell parameters from 1492 reflections
*a* = 8.3412 (8) Å	θ = 3.0–28.0°
*b* = 9.6501 (9) Å	µ = 0.43 mm^−^^1^
*c* = 9.5485 (9) Å	*T* = 293 K
β = 91.319 (9)°	Rod, colourless
*V* = 768.39 (13) Å^3^	0.40 × 0.20 × 0.20 mm
*Z* = 2	

### Data collection

**Table:**

Oxford Diffraction Xcalibur (TM) Single Crystal X-ray Diffractometer with Sapphire CCD Detector.	1535 independent reflections
Radiation source: fine-focus sealed tube	1253 reflections with *I* > 2σ(*I*)
graphite	*R*_int_ = 0.009
Rotation method data acquisition using ω scans.	θ_max_ = 26.4°, θ_min_ = 3.0°
Absorption correction: multi-scan (*CrysAlis RED*; Oxford Diffraction, 2009)	*h* = −10→4
*T*_min_ = 0.847, *T*_max_ = 0.919	*k* = −12→11
2574 measured reflections	*l* = −10→11

### Refinement

**Table:**

Refinement on *F*^2^	Primary atom site location: structure-invariant direct methods
Least-squares matrix: full	Secondary atom site location: difference Fourier map
*R*[*F*^2^ > 2σ(*F*^2^)] = 0.037	Hydrogen site location: inferred from neighbouring sites
*wR*(*F*^2^) = 0.105	H atoms treated by a mixture of independent and constrained refinement
*S* = 1.07	*w* = 1/[σ^2^(*F*_o_^2^) + (0.0482*P*)^2^ + 0.3792*P*] where *P* = (*F*_o_^2^ + 2*F*_c_^2^)/3
1535 reflections	(Δ/σ)_max_ = 0.002
103 parameters	Δρ_max_ = 0.25 e Å^−^^3^
1 restraint	Δρ_min_ = −0.34 e Å^−^^3^

### Special details

**Table:**

Experimental. Empirical absorption correction using spherical harmonics, implemented in SCALE3 ABSPACK scaling algorithm.
Geometry. All e.s.d.'s (except the e.s.d. in the dihedral angle between two l.s. planes) are estimated using the full covariance matrix. The cell e.s.d.'s are taken into account individually in the estimation of e.s.d.'s in distances, angles and torsion angles; correlations between e.s.d.'s in cell parameters are only used when they are defined by crystal symmetry. An approximate (isotropic) treatment of cell e.s.d.'s is used for estimating e.s.d.'s involving l.s. planes.
Refinement. Refinement of *F*^2^ against ALL reflections. The weighted *R*-factor *wR* and goodness of fit *S* are based on *F*^2^, conventional *R*-factors *R* are based on *F*, with *F* set to zero for negative *F*^2^. The threshold expression of *F*^2^ > σ(*F*^2^) is used only for calculating *R*-factors(gt) *etc*. and is not relevant to the choice of reflections for refinement. *R*-factors based on *F*^2^ are statistically about twice as large as those based on *F*, and *R*- factors based on ALL data will be even larger.

### Fractional atomic coordinates and isotropic or equivalent isotropic displacement parameters (Å^2^)

**Table:**

	*x*	*y*	*z*	*U*_iso_*/*U*_eq_	
Cl1	0.01685 (8)	0.54218 (6)	0.32765 (6)	0.0639 (2)	
O1	0.4067 (2)	0.17690 (15)	0.16810 (13)	0.0521 (4)	
N1	0.3460 (2)	0.28337 (17)	−0.03736 (15)	0.0395 (4)	
H1N	0.362 (3)	0.279 (2)	−0.1207 (17)	0.047*	
C1	0.2803 (2)	0.40783 (19)	0.01300 (18)	0.0344 (4)	
C2	0.1918 (2)	0.4127 (2)	0.13434 (19)	0.0382 (4)	
H2	0.1761	0.3332	0.1873	0.046*	
C3	0.1276 (2)	0.5375 (2)	0.1747 (2)	0.0416 (5)	
C4	0.1467 (3)	0.6572 (2)	0.0987 (2)	0.0508 (5)	
H4	0.1024	0.7404	0.1281	0.061*	
C5	0.2335 (3)	0.6502 (2)	−0.0223 (2)	0.0520 (5)	
H5	0.2469	0.7298	−0.0757	0.062*	
C6	0.3006 (2)	0.5279 (2)	−0.0655 (2)	0.0429 (5)	
H6	0.3595	0.5253	−0.1470	0.052*	
C7	0.4051 (2)	0.17778 (18)	0.04063 (18)	0.0363 (4)	
C8	0.4738 (3)	0.0598 (2)	−0.04393 (19)	0.0481 (5)	
H8A	0.3934	0.0286	−0.1118	0.058*	
H8B	0.5648	0.0938	−0.0953	0.058*	

### Atomic displacement parameters (Å^2^)

**Table:**

	*U*^11^	*U*^22^	*U*^33^	*U*^12^	*U*^13^	*U*^23^
Cl1	0.0725 (4)	0.0602 (4)	0.0602 (4)	0.0010 (3)	0.0256 (3)	−0.0182 (3)
O1	0.0881 (12)	0.0448 (8)	0.0235 (7)	0.0190 (8)	0.0080 (6)	0.0013 (6)
N1	0.0599 (10)	0.0367 (9)	0.0220 (7)	0.0081 (8)	0.0057 (7)	0.0004 (6)
C1	0.0402 (9)	0.0326 (9)	0.0304 (9)	0.0015 (8)	−0.0024 (7)	−0.0012 (7)
C2	0.0457 (11)	0.0337 (10)	0.0353 (9)	−0.0009 (8)	0.0016 (8)	−0.0019 (7)
C3	0.0413 (10)	0.0433 (11)	0.0402 (10)	0.0002 (9)	0.0023 (8)	−0.0088 (8)
C4	0.0525 (12)	0.0367 (11)	0.0631 (14)	0.0083 (9)	0.0000 (10)	−0.0066 (10)
C5	0.0604 (13)	0.0362 (11)	0.0593 (13)	0.0035 (10)	−0.0001 (10)	0.0108 (10)
C6	0.0488 (11)	0.0425 (12)	0.0376 (10)	0.0030 (9)	0.0027 (8)	0.0067 (8)
C7	0.0507 (11)	0.0329 (9)	0.0254 (8)	0.0020 (8)	0.0054 (7)	−0.0010 (7)
C8	0.0784 (15)	0.0393 (11)	0.0266 (9)	0.0136 (10)	0.0052 (9)	−0.0022 (8)

### Geometric parameters (Å, °)

**Table:**

Cl1—C3	1.747 (2)	C4—C5	1.380 (3)
O1—C7	1.217 (2)	C4—H4	0.9300
N1—C7	1.349 (2)	C5—C6	1.373 (3)
N1—C1	1.409 (2)	C5—H5	0.9300
N1—H1N	0.811 (16)	C6—H6	0.9300
C1—C2	1.389 (3)	C7—C8	1.516 (3)
C1—C6	1.393 (3)	C8—C8^i^	1.487 (4)
C2—C3	1.377 (3)	C8—H8A	0.9700
C2—H2	0.9300	C8—H8B	0.9700
C3—C4	1.375 (3)		
			
C7—N1—C1	126.55 (15)	C6—C5—C4	121.3 (2)
C7—N1—H1N	116.0 (16)	C6—C5—H5	119.4
C1—N1—H1N	116.8 (16)	C4—C5—H5	119.4
C2—C1—C6	119.64 (18)	C5—C6—C1	119.87 (19)
C2—C1—N1	122.13 (16)	C5—C6—H6	120.1
C6—C1—N1	118.19 (17)	C1—C6—H6	120.1
C3—C2—C1	118.72 (18)	O1—C7—N1	123.58 (16)
C3—C2—H2	120.6	O1—C7—C8	122.13 (17)
C1—C2—H2	120.6	N1—C7—C8	114.28 (15)
C4—C3—C2	122.47 (19)	C8^i^—C8—C7	113.09 (19)
C4—C3—Cl1	119.31 (16)	C8^i^—C8—H8A	109.0
C2—C3—Cl1	118.22 (16)	C7—C8—H8A	109.0
C3—C4—C5	118.03 (19)	C8^i^—C8—H8B	109.0
C3—C4—H4	121.0	C7—C8—H8B	109.0
C5—C4—H4	121.0	H8A—C8—H8B	107.8
			
C7—N1—C1—C2	−35.0 (3)	C3—C4—C5—C6	−0.7 (3)
C7—N1—C1—C6	147.5 (2)	C4—C5—C6—C1	0.5 (3)
C6—C1—C2—C3	−0.7 (3)	C2—C1—C6—C5	0.2 (3)
N1—C1—C2—C3	−178.16 (17)	N1—C1—C6—C5	177.74 (19)
C1—C2—C3—C4	0.6 (3)	C1—N1—C7—O1	1.0 (3)
C1—C2—C3—Cl1	−179.87 (14)	C1—N1—C7—C8	−177.66 (19)
C2—C3—C4—C5	0.1 (3)	O1—C7—C8—C8^i^	5.9 (4)
Cl1—C3—C4—C5	−179.44 (17)	N1—C7—C8—C8^i^	−175.4 (2)

Symmetry codes: (i) −*x*+1, −*y*, −*z*.

### Hydrogen-bond geometry (Å, °)

**Table:**

*D*—H···*A*	*D*—H	H···*A*	*D*···*A*	*D*—H···*A*
N1—H1N···O1^ii^	0.81 (2)	2.10 (2)	2.8946 (19)	166 (2)

Symmetry codes: (ii) *x*, −*y*+1/2, *z*−1/2.
